# Academy of Medicine in Ireland

**Published:** 1883-09

**Authors:** 


### Academy of Medicine in Ireland.

### SURGICAL SECTION.

At the closing meeting of the past session, Mr. J. K. Barton, President, in the chair, Dr. Theodore Stack read a paper on "The Replantation and Transplantation of Teeth." After briefly referring to the history of the operation, Dr. Stack went on to speak of the more recent experiences of Mr. Coleman, Dr. Magitot, and others. He believed that Mr. Coleman's plan of operating antiseptically was correct in theory, but that it failed in practice because he used the carbolic acid in too concentrated a form. He referred also to the papers read by Drs. Magitot and Finlay Thompson before the Dental Section of the International Medical Congress.

Replantation would be found a useful therapeutic measure under the following circumstances:

1. In cases where the pulp was exposed, or nearly exposed, and a carious cavity extended under the gum.
2. When teeth had been knocked out by external violence.
3. After accidental extraction.
4. In obscure cases of neuralgia referred to sound teeth.
5. In cases of alveolar abscess, complicated or uncomplicated.

The last named was the most common cause for which the operation was undertaken. He exhibited a table of some thirty cases in which the operation had been performed by Mr. A. W. Baker and himself, both in private and hospital practice; all of them had proved successful. In most the treatment had been resection of the root, filling the canal with creosote and iodoform, and free incision into the alveolar abscess. Referring to the liability of these teeth to absorption, a danger mentioned by Tomes, Coleman and other authorities, Dr. Stack said he believed this danger only applied to teeth which had been so treated whilst out of the mouth as to cause death of the periodontal membrane, either by too long delay, or by the use of some too strong chemical agent. It was not due to rending of the alveolar connections, for teeth violently knocked out and quickly replanted nearly always became firm and useful. Nor was it due to filling the pulp chambers and canals with foreign material, for in cases of pivoting it was no uncommon occurrence for the root to last twenty or thirty years. In the Museum of the Dental Hospital of Ireland was a specimen of a pivot tooth, presented by Mr. Daniel Corbett, which had lasted thirty-seven years. In the allied operation of transplantation, in which the scion tooth was always perfect, it was still undecided whether the pulp should be exterminated or not. He believed that Mr. A. W. Baker, Mr. Abraham and himself were the first who had established, by actual microscopical examination in the human subject, that the pulp chamber in the scion tooth could after replantation again enclose living contents; but though this was a possible, perhaps a probable, result, it was by no means a universal one. He believed that the operation of transplantation was likely to grow in favor, especially in hospital practice, where the patients were unable to pay for good artificial dentures.

In the course of the discussion which followed, Dr. R. McDonnell said that, with reference to the cases spoken of by Dr. Stack, he should be very cautious in drawing the conclusion that the substance found in the pulp cavity of the replanted tooth was actually revived pulp. It might be that granulations had sprung up and filled the cavity, that the tooth was acting like a sponge graft, and was therefore in the happy position of having a pulp without any nerve in it.

An illustration of stinginess is cited by an Arkansas editor, who knows a man that talks through his nose in order to save wear and tear on his false teeth.

